# Literary Notes

**Published:** 1898-06

**Authors:** 


### LITERARY NOTES.

THE *New Hampshire Sanitary Journal* made its appearance during May, 1898. It is a quarterly magazine, published in the interest of healthful homes and communities, is the official organ of the New Hampshire board of health and of the New Hampshire association of boards of health. It is edited by Dr. Irving A. Watson and is published at Concord, New Hampshire. The first number contains 64 pages, is full of interesting material and is handsomely printed on antique paper. Its subscription price is $1.00 a year.

---

THE *International Medical Magazine* has been published since January 1, 1898, under new editorial and business management. The *Magazine* has been considerably enlarged, is especially illustrated, several new and attractive departments have been added, and the subscription price has been reduced to ($2.00) two dollars a year. Nevertheless, the magazine retains its high standard and is one of the best medical monthlies published.

---

SAJOUS'S Annual and Analytical Cyclopedia of Practical Medicine, a review of the first volume of which will be found in its appropriate place in the JOURNAL, will appear at the approximate rate of one volume each six months, the whole alphabet being thus covered in three years, and during this time a monthly supplement (The Monthly Cyclopedia), alphabetical from A to Z, will be brought out, so that a doctor can have a complete synopsis of the latest journal literature to reinforce his system of reference.

Subscriptions are taken for the entire series only, at $5.00 a volume, in cloth. This secures a large six-volume reference system with thirty-six monthly supplements during that period.

The volumes will be beautifully illustrated; each volume will be handsomely bound in two colors of cloth. The half Russia binding will be furnished at $6.00 a volume, if desired.

---

THE *Memphis Lancet* will, it is announced, begin publication on July 1st, 1898, as a monthly journal devoted to progressive medicine. It is the purpose of the editors to put it in the front rank of medical periodicals, and to that end they assert that they will spare neither time, pains nor money. It will be published on the first of each month, and will contain fifty-four pages of reading matter, including original articles, editorials, news and notes and abstracts from other publications, giving a full résumé of the progress of medical science. We wish the *Lancet* a full measure of success.

---

THE *Laryngoscope*, St. Louis, beginning with the July, 1898, issue (Vol. VI., No. 1,) proposes to publish a complete bibliography and abstract department containing a résumé of the entire field of otology, rhinology and laryngology, as gleaned from the pages of the medical journal press from every section of the world. The enterprise is commendable.

---

THE *National Medical Review*, beginning with its March issue, publishes a supplement called The Military Surgeon, which is under the charge of Colonel William H. Forwood, M. D., assistant surgeon-general United States army. The first article in the May issue is entitled

Field service, by George W. Adair, M. D., assistant surgeon U. S. army, and should be read by every medical officer of the National Guard now entering the United States service. Following this are several pages of well-selected material from home and foreign journals relating to military medicine and surgery.

---

THE *American Journal of Insanity*, one of the ablest and staunchest of medical magazines, has for the past year been published under the auspices of the American medico-psychological association with the following board of editors: Drs. Edward N. Brush, G. Alder Blumer and J. Montgomery Mosher, with Henry M. Hurd as editor-in-chief. Its place of publication has been changed to Baltimore, and is published by the Johns Hopkins Press.

---

THE Buffalo general hospital has issued its thirty-ninth annual report, which covers the year 1897. During the year 2,050 cases were treated, of which 806 were medical, 841 surgical, 294 gynecological, 83 obstetrical and 26 were ophthalmological patients. The number of deaths during the year was 213, and 702 ambulance calls were answered. The number of pupils graduated in the training school was 13. It is needful that the new building should be completed, as the demands upon the hospital are in excess of its capacity. Here is an opportunity for philanthropists.

---

BRIGHAM HALL, a hospital for the insane, located at Canandaigua, is one of the best private institutions in the country for patients afflicted with nervous and mental diseases. Its location is most healthful, the water supply ample and pure and its environment is charming. It is under the charge of Dr. D. R. Burrell, who is an accomplished alienist and an able administrator. The hospital has issued an interesting illustrated circular, in which it states that cases of neurasthenia and of the alcohol or drug habit, who are not insane, but are willing to conform to the regulations of the hospital and to the advice of the physicians, will be received on voluntarily signing a request for admission.

---

THE state board of health of Massachusetts has issued an elaborate report on Epidemic cerebro-spinal meningitis and its relation to other forms of meningitis. This is an illustrated book of 178 pages and is a credit to the board from a scientific standpoint, and a work in which neurologists will be interested.

